# Surface-Initiated Atom Transfer Polymerized Anionic Corona on Gold Nanoparticles for Anti-Cancer Therapy

**DOI:** 10.3390/pharmaceutics12030261

**Published:** 2020-03-13

**Authors:** Wei Mao, Sol Lee, Ji Un Shin, Hyuk Sang Yoo

**Affiliations:** 1 Department of Biomedical Materials Engineering, Kangwon National University, Chuncheon 24341, Korea; maowei@kangwon.ac.kr (W.M.); dlthf789@kangwon.ac.kr (S.L.); jiwun77@gmail.com (J.U.S.); 2 Institute for Molecular Science and Fusion Technology, Kangwon National University, Chuncheon 24341, Korea

**Keywords:** surface-initiated atom transfer radical polymerization, gold nanoparticle, c-Myc siRNA, anti-cancer therapy

## Abstract

Surface initiated atom transfer radical polymerization (SI-ATRP) documented a simple but efficient technique to grow a dense polymer layer on any surface. Gold nanoparticles (AuNPs) give a broad surface to immobilize sulfhyryl group-containing initiators for SI-ATRP; in addition, AuNPs are the major nanoparticulate carriers for delivery of anti-cancer therapeutics, since they are biocompatible and bioinert. In this work, AuNPs with a disulfide initiator were polymerized with sulfoethyl methacrylate by SI-ATRP to decorate the particles with anionic corona, and branched polyethyeleneimine (PEI) and siRNA were sequentially layered onto the anionic corona of AuNP by electrostatic interaction. The in vitro anti-cancer effect confirmed that AuNP with anionic corona showed higher degrees of apoptosis as well as suppression of the oncogene expression in a siRNA dose-dependent manner. The in vivo study of tumor-bearing nude mice revealed that mice treated with c-Myc siRNA-incorporated AuNPs showed dramatically decreased tumor size in comparison to those with free siRNA for 4 weeks. Furthermore, histological examination and gene expression study revealed that the decorated AuNP significantly suppressed c-Myc expression. Thus, we envision that the layer-by-layer assembly on the anionic brushes can be potentially used to incorporate nucleic acids onto metallic particles with high transfection efficiency.

## 1. Introduction

Surface initiated atom transfer polymerization (SI-ATRP) was employed to decorate surfaces with dense layers of polymers to change the surface chemistry with desirable features [1,2]. In comparison to conventional surface-immobilization of polymeric chains, densely-packed brushes of oligomeric chains can be easily synthesized for an average thickness of 58~72 nm with halide-initiated propagation of monomers, which is sufficient to manipulate surface characteristics with certain monomers [3]. Brushes on flat surfaces can be fabricated using both the ‘‘top down’’ and the ‘‘bottom up’’ approaches, because the nanoscale organization and the functionality of tethered polymers can be manipulated by techniques such as photolithography and micro- and nanocontact printing. The synthesis of brushes on particle surfaces has been widely conducted to prepare solid supports, chromatographic stationary phases, and high surface area models for brushes on flat surfaces. SI-ATRP is a typical “bottom up” technique that is universally employed to decorate various surfaces with polymers, including organic latex colloid emulsions, shells of shell-crosslinked micelles, and inorganic particles, such as silica, gold, alumina, polysilsesquioxane, titanium oxide clusters, iron oxides, and germanium [4].

Although anti-cancer siRNA has been extensively explored to silence oncogenic expression with high efficiency, the lack of proper carriers to deliver the cargo has frequently impeded successful suppression of the targeted oncogenes because of insufficient stability and non-specificity of siRNA [5,6]. Because the structures of siRNA are short and rigid, they can be easily degraded, and siRNA needs flexible carriers for gene delivery. This carrier can prevent degradation from RNase. According to a previous study, to overcome the limitation of siRNA, mono-siRNA and poly-siRNA (thiolated siRNA) were complexed with polyethyeleneimine (PEI) for stable gene delivery [7]. Consequently, poly-siRNA/PEI complexation was found to be superior in siRNA protection than the mono-siRNA/PEI complexation. However, after complexation with PEI, although single siRNA demonstrated worse stability than poly-siRNA, it showed preserved activity in comparison to naked siRNA after a long incubation time in physiological conditions [8]. In addition, stability of siRNA could be further enhanced by incorporation of siRNA to a cationic polymer-immobilized solid nanoparticle [9]. Commonly, siRNA delivery has been studied by complexation with PEI because PEI has a flexible structure and can be easily used. This facilitates PEI@siRNA nanoparticles, in particular, to be studied repeatedly. However, PEI has light cytotoxicity, and it is very difficult to visualize in an in vivo system for determining circulation in the body system. However, gold nanoparticles (AuNPs) have advantages of small size, non-cytotoxicity, non-immunogenicity, and the possibility of being used in imaging; however, they have the disadvantage of a rigid structure. Therefore, they are not appropriate for siRNA delivery as the flexible carrier; however, their surfaces can be modified by polymers using several types of methods. Among these methods, ATRP can be used to form a flexible polymer layer and regulate the length of polymer brushes. Unlike directly immobilizing polymers on a surface, which usually results in a loosely arranged polymer layer, initiators with small molecular weight can be densely arranged on a surface to polymerize monomers into a dense polymer layer because the steric hindrance is weaker among small initiators and monomers than among high molecular-weight polymers.

To employ AuNPs as imaging and drug delivery carriers, surface-decoration of AuNP was performed to engineer AuNPs with tailor-made features, such as specific charges, ligands, and functional groups. For this purpose, SI-ATRP was employed to construct specific corona with a high surface density. According to previous studies about SI-ATRP on the surfaces of AuNPs, AuNPs were synthesized with three layers of poly(2-(dimethylamino) ethyl methacrylate-2-hydroxyethyl methacrylate copolymers using ATRP, and siRNA was incorporated for gene delivery. Owing to ATRP, the surface of the AuNP was densely decorated with polymeric brushes, and the efficacy of incorporation of siRNA was enhanced [9]. To obtain enhanced surface-functionalized gold nanoparticles, sugar methacrylate was polymerized on the surface of AuNPs using ATRP. These particles have enhanced cell recognition, cell adhesion, and cell growth regulation because of glycoconjugates by SI-ATRP [10]. Because of the superior biocompatibility of AuNP/silica hybrids nanoparticles, these particles were decorated with 2-(dimethylamino)ethyl methacrylate (DMAEMA) using ATRP for the bioprobes and the sensors [11]. They combined surface-initiated polymerization and biosilicification to prepare AuNP/silica core/shell hybrid nanoparticles by biomimetic silicification of silicic acids with poly(DMAEMA). The decorated hybrids presented several advantages, such as rapid measurement and being relatively easy to use. Further, they presented the possibility of atomic force microscopy (AFM) scanning for measurement of surface thickness. Therefore, they could be used in full indentation methods for measuring the thickness [12].

In the current study, we prepared AuNPs with surface decorated poly(sulfoethyl methacrylate) (SEMA@AuNP) using ATRP for gene delivery for anti-cancer effects. Branched PEI (PEI) and siRNA were incorporated using electrostatic interaction between the negatively charged SEMA (PEI@SEMA@AuNP) and the positively charged PEI (siRNA@PEI@SEMA@AuNP), respectively. The SI-ATRP of a dense anionic polymer layer on the AuNPs surface enhanced the surface negative charge and thus elevated the subsequent decoration amount of PEI, which further increased the siRNA sequester efficiency in comparison to siRNA incorporation efficiency on PEI-modified bare AuNPs. Surface analysis of decorated particles as well as in vitro and in vivo tests were performed to determine the efficiency of these particles.

## 2. Materials and Methods

### 2.1. Materials

Gold(III) chloride trihydrate (HAuCl_4_), trisodium citrate dehydrate, bis[2-(2’-bromoisobutryloxy)ethyl]disulfide, 2,2’-bipyridyl (bpy), copper (I) bromide (CuBr(I)), and copper (II) bromide (CuBr (II)) were purchased from Sigma Aldrich (St. Louis, MO, USA). The 2-Sulfoethyl methacrylate (SEMA) and the polyethylenimine (PEI) (branched, Mw 10,000) were obtained from Polysciences Inc. (Warrington, PA, USA). Alexa Fluor 647-NHS ester and a LIVE/DEAD assay kit for mammalian cell were purchased from Thermo Fisher Scientific (Waltham, MA). RPMI 1640, phosphate buffered saline (PBS), fetal bovine serum (FBS), and penicillin/streptomycin were purchased from Thermo Fisher Scientific. Adenocarcinomic human alveolar cell line (A549) was purchased from ATCC (Manassas, VA, USA). An RNA extraction kit (Hybrid-RTM) was purchased from GeneAll^®^ (Seoul, Korea). Propidium iodide (PI) was purchased by Invitrogen (Carlsbad, CA, USA). Hipi Plus 5× PCR Premix, Reverse Transcription Premix (5×, 20 μL reaction M-MLV-RT, RNase H+), and HiPi Real-Time PCR 2× Master Mix were purchased from Elpis Biotech (Daejeon, Korea). siRNA was synthesized from Bioneer (Daejeon, Korea). Anti-c-Myc primary antibody for tumor tissue staining was purchased from Santa Cruz Biotechnology Inc. (Dallas, TX, USA). EzWayTM DAB Substrate Kit and R.T.U. VECTASTAIN^®^ Universal ABC Kit were purchased from Koma Biotech Inc. (Seoul, Korea), Vector Laboratories, Inc. (Burlingame, CA, USA), respectively. Hematoxylin was purchased from YD Diagnostics (Yongin, Korea).

### 2.2. Synthesis of Gold Nanoparticles (AuNPs) and Surface Decoration by ATRP

AuNPs were fabricated via reduction of gold chloric acid and subsequently surface-modified with polymethacrylate derivatives as previously described with a minor modification [9]. After, HAuCl_4_ (1 mM) solution was heated to 90 °C, trisodium citrate solution (38.8 mM) was added, and the solution was stirred for 10 min to obtain AuNPs. For ATRP, bis[2-(2’-bromoisobutryloxy)ethyl] disulfide (disulfide initiator, 0.88 mg; molar ratio of AuNP:initiator = 1:5) in dimethtyl formamide (DMF) was added dropwise to 10 mL of AuNP (1 mg) with stirring and further incubated for 12 h. The unreacted initiator was subsequently removed by centrifugation at 15,520× *g* for 10 min (3 times) and washed with DMF. Surface initiated AuNPs were slowly added to a solvent mixture (DMF:distilled water:MeOH = 2:1:5 (*v*/*v*), 10 mL) containing bpy and SEMA monomers with nitrogen purging. After 15 min, CuBr(I) in DMF was added and the reaction mixture was further incubated at 25 °C with vigorous stirring for 6 h (molar ratio of initiator on AuNP: monomer: CuBr(I):bpy = 1:100:1:2 = 0.88 mg:23 mg:0.17 mg:0.374 mg). To slow down the polymerization process, CuBr(II) was also added at 10% of CuBr(I). To characterize surface immobilized polymer layers on AuNPs, hydrodynamic size and surface charge were measured using dynamic light scattering and a ζ-potential analyzer (Zetasizer Nano, Malvern Instrument). Surface plasmon resonance (SPR) of the nanoparticles was also monitored from 200 to 800 nm (6505 UV/Vis. Spectrophotometer, Jenway). To determine the polymerization degree of the surface polymerized SEMA (pSEMA), the SEMA functionalized AuNPs (SEMA@AuNPs) were coated on an ɑ-cyano-4-hydroxycinnamic acid (CHCA) matrix and analyzed using matrix-assisted laser desorption ionization time-of-flight (MALDI-TOF) mass spectroscopy with 20 kV of accelerated voltage (Bruker Autoflex, Bruker). For visualizing the polymer decoration on the surface of AuNPs, the surface of SEMA@AuNPs was elementally mapped using an energy filtering transmission electron microscope equipped with an energy dispersive spectrometer (LEO 912AB OMEGA) to detect nitrogen (N) of PEI and phosphate (P) of siRNA layers on the AuNPs.

### 2.3. PEI Decoration on SEMA@AuNPs

To decorate SEMA@AuNPs with PEI, PEI was electrostatically incorporated onto SEMA@AuNP, and the binding behaviors were characterized. Briefly, PEI (5 μg) was incubated with SEMA@AuNPs (100 μg) in PBS for 3 h. After incubation, PEI decorated SEMA@AuNPs (PEI@SEMA@AuNPs) were washed with PBS via centrifugation at 12,000 rpm for 10 min. Bare AuNP with the same amount of PEI (PEI@AuNPs) was employed as a control. To obtain PEI release profiles from AuNPs, Alexa Fluor 647 conjugated PEI was alternatively employed, and the respective AuNPs (PEI@SEMA@AuNPs or PEI@AuNPs) were incubated in PBS at 37 °C with gentle shaking. Alexa Fluor 647 NHS ester was conjugated to the primary amines of PEI according to manufacturer’s protocol (molar ratio of Alexa Fluor 647 NHS ester:PEI = 1:1). The supernatant was collected, and the fluorescence intensity was measured (λ_excitation_: 650 nm, λ_emission_: 666 nm).

### 2.4. Incorporation of siRNA on PEI@SEMA@AuNPs

Various amounts of siRNA were electrostatically loaded in PEI@SEMA@AuNP, and the incorporation behaviors of siRNA were investigated. siRNA was incorporated on the surface of PEI@SEMA@AuNPs, and the sizes and the surface charges were measured as mentioned previously with brief modifications [13,14,15]. Briefly, various amounts of siRNA (0.625 μg, 1.25 μg, and 2.75 μg) in PBS (0.1 mL) were slowly added to PEI@SEMA@AuNPs (50 μg) in PBS (0.9 mL) and further incubated at 37 °C with gentle stirring for 6 h. The siRNA-incorporated particles (siRNA@PEI@SEMA@AuNPs) were centrifuged at 12,000 rpm, and the absorbance of the supernatant was measured at 260 nm to quantify the amount of unconjugated siRNA. siRNA conjugated PEI@SEMA@AuNPs were also characterized using dynamic light scattering to determine the size and the ζ-potential (Zetasizer Nano, Malvern Instrument, Malvern Instrument, Malvern, UK). Two micrograms of siRNA conjugated PEI@SEMA@AuNPs were analyzed via electrophoresis with 0.8% (*w*/*v*) agarose gel and visualized using a UV transilluminator.

### 2.5. In Vitro Anti-Cancer Effect

The anti-cancer effects of siRNA-incorporated AuNPs were quantified and visualized using an 3-(4,5-dimethylthiazol-2-yl)-2,5-diphenyl tetrazolium bromide (MTT)-based cytotoxicity assay and a LIVE/DEAD assay as previously described with a minor modification [16]. Briefly, A549 cells were cultivated in RPMI 1640 supplemented with 10% FBS and 1% penicillin/streptomycin in a 5% CO_2_ humidified incubator when they reached a confluency of 70%. For MTT-based cytotoxicity assay, cells (5.5 × 10^4^ cells/well) were harvested and seeded in the 24 wells. After 12 h, siRNA-incorporated AuNPs (siRNA equivalent = 0.5~2.5 μg/well) were added to each well. After 3 h, the cell culture medium was removed, and MTT reagent (40 μL, 5 mg/mL) was added to each well containing cell culture medium (400 μL) and further incubated for 3 h. Formazan crystal was dissolved with dimethyl sulfoxide (DMSO), and the absorbance was measured at 570 nm. For the LIVE/DEAD assay, cells were incubated with each sample for 5 h and then stained with 4 μM EthD-1 and 2 μM calcein AM in the PBS for 30 min. Fluorescently-labeled cells were visualized with a fluorescence microscope using filter sets of red and green lights (Eclipse Ti, Nikon Inc., Tokyo, Japan). To quantify the dead cells, the red cells in fluorescence images were counted, and the ratio was calculated with respect to total number of cells in the image (ca. 100 cells/image).

### 2.6. siRNA Silencing of c-Myc Gene

To assess the effect of siRNA-incorporated AuNP for c-Myc suppression, A549 cells were used to quantify gene silencing levels using quantitative RT-PCR (qRT-PCR) as described previously [16]. Cells in a 24 well plate (5.4 × 10^4^ cells/well) were treated with free siRNA, siRNA incorporated PEI@AuNPs, and PEI@SEMA@AuNPs for 3 h, and total RNA was extracted with an RNA extraction kit^®^ reagent according to manufacturer’s protocol. cDNA was synthesized at 45 °C using a cDNA synthesis kit (HiPi Plus 5x PCR Master Mix, ElpisBio, Daejeon, Korea). qRT-PCR was performed with the followed conditions; 38 cycles at 95 °C for 30 s, 57 °C for 30 s, and 72 °C for 30 s for GAPDH; 38 cycles at 95 °C for 30 s, 55 °C for 30 s, and 72 °C for 30 s for c-Myc. The GAPDH and the c-Myc primer sequences used for qPCR were (forward primer) 5′-ACC CAG AAG ACT GTG GAT GG-3′, (reverse primer) 5′-TTC TAG ACG GCA GGT CAG GT-3′, c-Myc primer: forward primer: 5′-TCA AGA GGC GAA CAC ACA AC-3′, and reverse primer: 5′-GGC CTT TTC ATT GTT TTC CA-3′, respectively; these were previously designed [9]. The gene expression level was normalized to 2^(−ΔΔ*C*_t_), where Δ*C*_t_ refers to the cyclic threshold (*C*_t_) value of the target gene—*C*_t_ value of the housekeeping gene.

### 2.7. Apoptotic Cell Death

To investigate the anti-cancer mechanism of siRNA-incorporated AuNPs, apoptotic cell death was monitored by tracking cell cycle arrest. A549 cells (1 × 10^5^ cells) were seeded and incubated in a 6-well plate for 12 h at 37 °C, 5% CO_2_. Prepared AuNPs (2.5 μg siRNA/50 μg AuNP, free siRNA, AuNP, SEMA@AuNP, PEI@SEMA@AuNP, PEI@AuNP, siRNA@PEI@SEMA@AuNP, siRNA@PEI@AuNP) were treated with the cells for 6 h in serum free media. The cells were washed with PBS thrice and further incubated with serum containing media for 48 h. The cells were trypsinized and fixed by 70% EtOH at −20 °C for 12 h. After being fixed with 2.5% glutaldehyde solution, the cells were permeabilized with 0.2% Triton X-100 in ice for 15 min. The cells were then briefly washed and stained with PI for 30 min and analyzed using flow cytometry. Stained cells were detected in FL-2 channels ((λ_excitation_: 535 nm, λ_emission_: 617 nm), and the gated events (10,000 events per sample) were analyzed using CellQuest software, where the histograms of FL-2 levels vs. events were obtained, and their phases were separated into 4 distinctive phases according to their DNA contents (sub G1, G0/G1, S and G2/M) as previously described (FACScalibur flow cytometer, BD Biosciences) [17].

### 2.8. Tumor Model and siRNA Treatment for Anti-Cancer Activity and Imaging

For tumor induction, A549 cells (1 × 10^7^ cells/100 μL PBS/injection) were subcutaneously injected into the right and the left flanks of female BALB-c/nude mice aged 4 weeks (Daihan Biolink, Korea) [16]. When the volume of the solid tumor reached 40–50 mm^3^/tumor, naked c-Myc siRNA, bare AuNP, siRNA@PEI@AuNP, and siRNA@PEI@SEMA@AuNP suspended PBS (100 μL) and a control group (PBS, 100 μL) were intravenously injected through the tail vein on days 1, 4, 7, 10, and 13 (2.5 μg siRNA/injection). The body weight and the tumor size of the mice were measured every 2 days, where the tumor volume was determined by (major axis) × (minor axis)^2^/2. For in vivo live imaging of animals, AF647-siRNA and AF647-siRNA@PEI@SEMA@AuNP (100 μg of siRNA) were intravenously injected. At 0 h and 12 h post-injection, the mice were anesthetized and fluorescently scanned using VISQUE In Vivo Smart (Vieworks Co., Korea) with a Cy5.5 filter set (λ_emission_: 630~680 nm; λ_emission_: 690~740 nm) at the Korea Basic Science Institute (KBSI) with 3 s of exposure time, and the fluorescent intensity was analyzed (CleVue Version 2.1.3.540, Vieworks Co.). All animal experiments were conducted in accordance with the institutional guidelines for the care and use of experimental animals at Institutional Animal Care and Use Committee of Kangwon National University (KW-180525-1, 2018-08-20).

### 2.9. c-Myc Expression in the Tumor Tissue

After harvesting tumors from the tumor-bearing mice at 28 days, the tumors were minced and homogenized with an RNA extraction reagent (100 mg/mL) for 2 days at 4 °C. The total RNA was extracted according to the manufacturer’s protocol. Furthermore, cDNA synthesis and RT-PCR were performed by the same method as that used in vitro. To determine the relative gene expression level, c-Myc and GAPDH primers were used and measured. The c-Myc primer sets were as follows: forward primer 5′–CG GAC ACA CAA CGT CTT GGA A-3′, and reverse primer 5′–AG GAT GTA GGC GGT GGC TTT T-3′; these sets were designed previously [18].

### 2.10. Immunohistochemical Staining from the Tumor Tissue

For visualizing the protein expression level of c-Myc at the tumor tissue, immunohistochemistry (IHC) staining was performed using c-Myc antibody and hematoxylin for counterstaining. The harvested tumors were fixed at 4% paraformaldehyde overnight at 4 °C and washed by PBS. After being dehydrated with graded ethanol for 1 h at 4 °C, the tumor tissue was immersed in xylene and subsequently embedded in paraffin. The paraffin block was cut into sections with 4 μm-thickness and deparaffinized using xylene and graded ethanol (100, 70, 30%). The tissue section was treated with 0.3% H_2_O_2_ in PBS for 30 min and diluted horse serum for 20 min to deactivate the peroxidase and prevent non-specific staining. The c-Myc primary antibody was treated to the tissue section at 4 °C for 12 h and the secondary antibody, Ready-to-Use Peroxidase (R.T.U ABC) reagent, and 3,3′-Diaminobenzidine (DAB) substrate solution were added and incubated for 30 min, respectively. The nucleus was counter-stained with hematoxylin for 5 min.

### 2.11. Statistical Analysis

Unless otherwise specified, all experiments were performed in triplicate. Statistical analysis was determined by one-way ANOVA with Sigma Plot v9.0 software, and *p* < 0.05 was considered statistically significant.

## 3. Results and Discussion

### 3.1. Preparation of SEMA@AuNP, PEI@SEMA@AuNP, and siRNA@PEI@SEMA@AuNP

**Figure 1:**
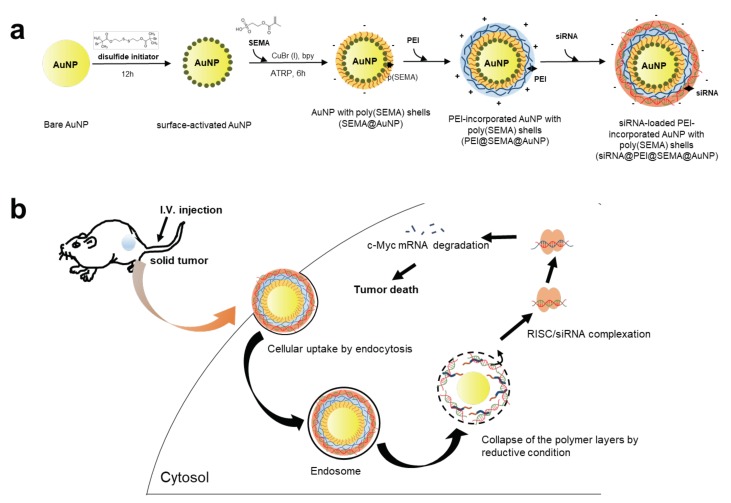
Schematic presentation of preparing and administration of AuNP with siRNA corona for suppression of solid tumors. (**a**) AuNP was anionically modified with surface initiated atom transfer radical polymerization (ATRP) of poly(sulfoethyl methacrylate) (SEMA) (SEMA@AuNP), electrostatically and sequentially incorporated with polyethyeleneimine (PEI) and siRNA (PEI@SEMA@AuNP), (siRNA@PEI@SEMA@AuNP). (**b**) In vivo administration of siRNA@AuNP for suppression of solid tumors.

To enhance gene silencing efficiency of siRNA with AuNP, we prepared siRNA-incorporated AuNP with SI-ATRP polymerized surface and tested the anti-tumor treatment in both in vitro and in vivo systems (Figure 1). Figure 1a shows the surface-decoration chemistry for (1) SI-ATRP of pSEMA on AuNPs (SEMA@AuNP) and (2) layer-by-layer assembly of PEI and siRNA. First, AuNPs were synthesized by adding the tri-sodium citrate as a reducing and capping reagent, and the surface citrate on the AuNP was substituted with disulfide initiator to polymerize sulfoethyl methacrylate monomer on the AuNP (SEMA@AuNP). Therefore, the surface charge of SEMA@AuNP was controlled by manipulating the monomer for SI-ATRP because the outmost layers of AuNP were completely shielded by the high density of pSEMA [19]. Although AuNP has been widely employed as a gene carrier, proper surface-decoration strategies have always received significant attention because siRNA must be decorated on the surface, unlike polymeric gene carriers encapsulating nucleic acids within the carriers. Thus, the surface must be carefully designed to ensure that incorporation and release of nucleic acids can be facilitated for successful gene transfer. We herein introduced a high density of anionic polymers on AuNP to enable further layer-by-layer incorporation of PEI and siRNA. Thus, PEI was electrostatically incorporated onto anionic corona of SEMA@AuNPs, and siRNA was incorporated into the PEI@SEMA@AuNP to prepare siRNA@PEI@SEMA@AuNP. Because PEI is widely used to deliver nucleic acid with high efficiency, the decorated AuNP is also anticipated to enhance the efficiency of gene delivery in the same manner that PEI enhances transfection [20]. After the endocytosis of AuNP with siRNA, the siRNA could be liberated by breaking the Au-S linkages in endosomes, which could liberate siRNA/PEI/SEMA shells from the AuNP. The siRNA could be further dissociated from PEI and escape from the endosomal region. The liberated siRNA in cytosol could inhibit the expression of c-myc by breaking down the RNA-induced silencing complex (RISC) for tumor suppression (Figure 1b).

### 3.2. Characterization of SEMA@AuNP, PEI@SEMA@AuNP and siRNA@PEI@SEMA@AuNP

#### 3.2.1. Size and Zeta Potential Measurement of AuNP, SEMA@AuNP, and PEI@SEMA@AuNP

**Figure 2:**
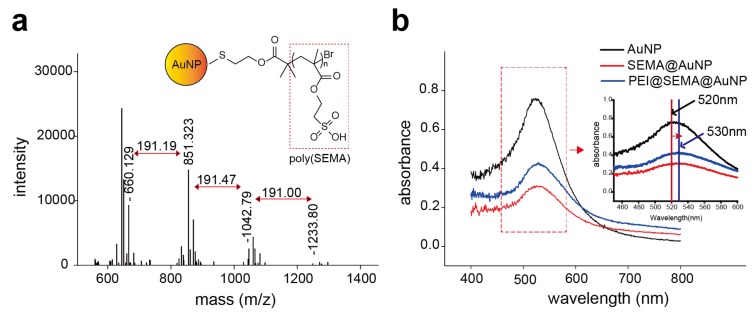
Characterization of surface-decorated AuNPs. (**a**) Quantification of immobilized pSEMA on the surface of particles by using MALDI-TOF/MS. (**b**) Absorbance of AuNPs and SEMA@AuNPs, PEI@SEMA@AuNPs. Absorbance was measured from 400 nm to 800 nm of wavelength. The absorbance of AuNPs had a red shift after modification with 10 nm wavelength. The inset shows a detail of the red shift with 520 nm (bare AuNP) to 530 nm (SEMA@AuNP).

After surface decoration of AuNP with pSEMA and PEI, we examined the particle characteristics to confirm the surface decoration, as shown in Table 1 and Figure 2. First, the hydrodynamic radius of the decorated particles dramatically increased upon SI-ATRP of pSEMA, where SEMA@AuNPs showed ca. 10-fold increase size increase compared to bare AuNP via dynamic light scattering (25.55 nm to 222.20 nm). This can be attributed to decoration of the hydrophilic chains composed of poly(methacrylate) derivatives on the solid particles, which can greatly increase hydrodynamic volume in the aqueous phase, as observed in our previous study [9]. Additionally, pSEMA decoration on AuNP leads to a larger increase in the hydrodynamic volume compared to the actual length of pSEMA oligomer because the hydorphilic pSEMA chains on AuNP increase the microviscosity around such particles [21]. Furthermore, because the surface stabilizer, citric acid was substituted with pSEMA, and decreased colloidal stability of the particles can affect such a large increase of the particle size. However, upon the electrostatic incorporation of PEI, PEI@SEMA@AuNP exhibited a smaller size compared with that of SEMA@AuNP (222.20 nm to 161.63 nm). The pSEMA on the surface could collapse because electrostatic repulsion among the anionic shells diminished due to the electrostatic binding of pSEMA to PEI. Compared to SEMA@AuNP, unmodified AuNPs, however, could not efficiently incorporate PEI because the diameter of PEI@AuNP was larger than that of PEI@SEMA@AuNP (180.63 ± 1.94 nm). Additionally, the surface charge of SEMA@AuNP changed from anionic to cationic upon PEI incorporation (−26.55 mV to 36.93 mV), thereby suggesting that PEI was covering SEMA@AuNP. However, PEI-coated AuNP (PEI@AuNP) exhibited a lower degree of cationic charges (14.37 ± 1.94 mV) compared to AuNP without anionic corona (PEI@SEMA@AuNP), which indicates that anionic shells composed of pSEMA on SEMA@AuNP allowed more PEI molecules to be electrostatically incorporated to AuNP. Accordingly, because SI-ATRP can produce densely-packed polymeric layers, we speculate that the anionic brushes on SEMA@AuNP efficiently incorporated PEI via electrostatic interactions [22,23].

#### 3.2.2. Characterization of pSEMA Presence and PEI Release from AuNPs

The immobilized anionic corona composed of pSEMA was further characterized by MALDI-TOF and UV/visible spectroscopic analysis (Figure 2). MALDI-TOF spectroscopy indicated that the major peaks of the molecular mass (*m*/*z*) of the polymers were 660, 851, 1042, and 1233, and the molecular mass difference among the peaks was 191, which corresponds to the molecular weight of the repeating unit. This clearly indicates that 4 SEMA was polymerized on the surfaces (Figure 2a). These results suggest that oligomeric layers of pSEMA covered the AuNPs and subsequently allowed them to have anionic corona on the surfaces. Additionally, plasmon resonance was observed at 520 nm for bare AuNP, however, SEMA@AuNP and PEI@SEMA@AuNP exhibited a peak absorbance at 530 nm, where a 10 nm red shift was observed (Figure 2b). Previous studies indicated that decoration of polymeric brushes caused 5~20 nm of red shift in the peak wavelength because of the change in plasmon resonance [9,24]. Thus, these results accordingly indicate that AuNP was successfully decorated with oligomeric pSEMA to form anionic brushes of polymeric corona. Additionally, we monitored the release of the incorporated PEI in PEI@SEMA@AuNP and PEI@AuNP to confirm that PEI can be tightly bound to SEMA@AuNP by electrostatic interactions (Figure S1). While PEI was gradually released from PEI@AuNP for 3 days (ca. 85%), no PEI release from PEI@SEMA@AuNP was observed for the same period (<1%). Thus, we speculate that PEI binding to SEMA@AuNP was significantly higher than that to AuNP because of the high density of anionic brushes on SEMA@AuNP. Because the surface charges of bare AuNP and SEMA@AuNP were not significantly different (−23.87 mV and −26.55 mV), densely packed and flexible brushes of anionic pSEMA corona on SEMA@AuNP are anticipated to greatly enhance PEI incorporation into the particle rather than the surface charge itself. We previously proposed that rough surfaces of cationic polymer brushes significantly enhance siRNA incorporation into the decorated surfaces [9]. Thus, the high yield of PEI incorporation on SEMA@AuNP is expected to show a high payload of siRNA in comparison to that of bare AuNP.

#### 3.2.3. Confirmation of siRNA Incorporation on PEI@SEMA@AuNPs

**Figure 3:**
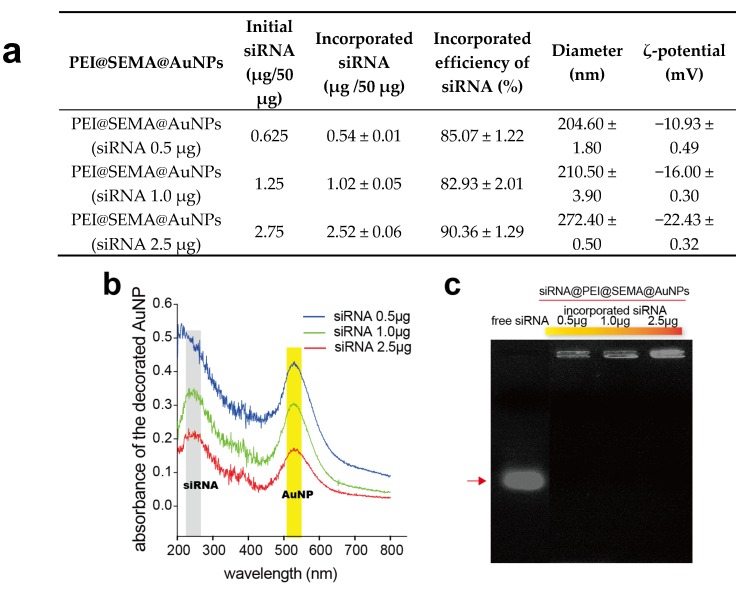
Characterization of siRNA incorporation in decorated AuNP. (**a**) siRNA incorporated AuNPs were characterized for incorporation efficiency, diameter, and surface charge. A fixed amount (50 μg) of PEI@SEMA@AuNP was electrostatically complexed with different amounts of siRNA. (**b**) UV/Vis spectrum siRNA@PEI@SEMA@AuNPs incorporating various amounts of siRNA (0.5~2.5 μg). The absorbance of siRNA presented at 260 nm of wavelength. (**c**) Gel retardation of PEI@SEMA@AuNPs incorporating various amounts of siRNA. Agarose gel electrophoresis was performed with 2 μg of siRNA equivalent at each well. The arrow indicates the location of the migrated free siRNA.

When various amounts of siRNA (0.625, 1.25, and 2.75 μg/50 μg) were incorporated to PEI@SEMA@AuNP, the resultant amounts of incorporated siRNA were confirmed to be 0.5, 1.0, and 2.5 μg, respectively (Figure 3a). The incorporation efficiencies did not significantly change among the samples (82~90%). However, the diameters and the ζ-potential showed significant changes depending on the amounts of incorporated siRNA, where the particles became larger and the surface charges gradually decreased. We speculate that higher incorporation of negatively charged siRNA to PEI@SEMA@AuNP allowed the particles to be more negatively charged, in addition to increasing the volume of hydrodynamic radius, because unique peaks of siRNA and AuNP appeared at 260 nm and 520 nm, respectively (Figure 3b). Additionally, the spectrum revealed that aggregation was not found during the electrostatic complexation process between siRNA and PEI@SEMA@AuNP because no red shift was observed. The gel retardation assay also confirmed that siRNA was completely incorporated in the particles with high efficiency because no visible band of siRNA was found in the gel except that of siRNA@PEI@SEMA@AuNP, which was retained in the well, where the intensity of staining became stronger because of escalating amounts of siRNA (Figure 3c) [9].

**Figure 4:**
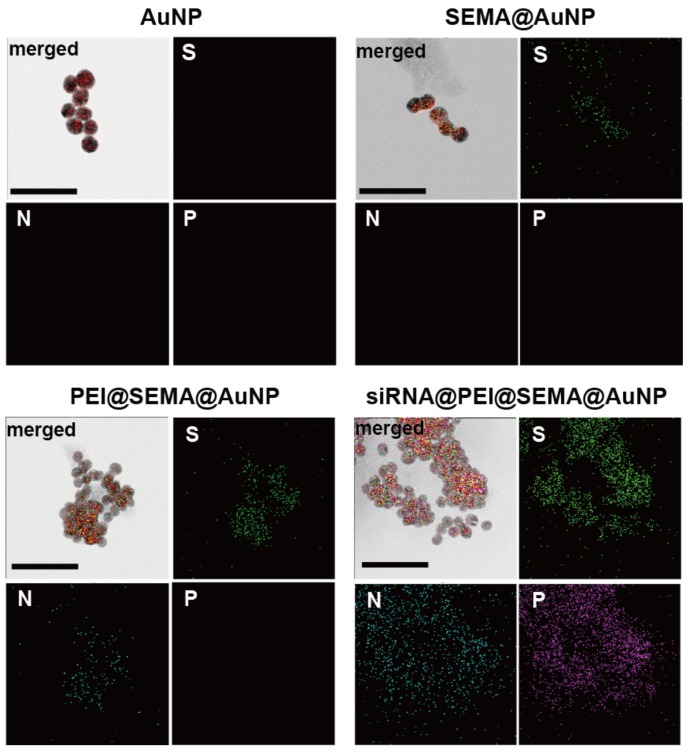
Elemental mapping images of surface modified AuNPs by TEM. Particles were visualized and elementally mapped for S, N, and P (green, blue, and purple, respectively). Merged images are TEM images overlapped with respective maps of respective atoms. siRNA@PEI@SEMA@AuNPs were loaded with 2.5 μg of siRNAs. (Scale bar = 50 nm).

Elemental mapping of the siRNA-incorporated particles using TEM also confirmed the incorporation of siRNA and PEI onto the surface-decorated AuNP (Figure 4). When sulfur (S), nitrogen (N), and phosphate (P) were observed by energy dispersive X-ray spectrometry (EDS), all AuNPs decorated with pSEMA exhibited distinct signals of S atom around the AuNP, which were caused by the disulfide initiator attached to the AuNP (SEMA@AuNP, PEI@SEMA@AuNP, and siRNA@PEI@SEMA@AuNP). However, the N atom was exclusively located in PEI-incorporated AuNP, while the P atom was only found in siRNA-incorporated AuNP, suggesting the proximity of PEI and siRNA onto the AuNP. Thus, these visualization results revealed that PEI and siRNA were electrostatically incorporated onto the anionic corona of the AuNP, as shown in Figure 1. However, although the SEMA was polymerized on the AuNP, these could not be detected because of the low degree of polymerization, which is characteristic of SI-ATRP, as shown in our previous work [9].

### 3.3. In Vitro Gene-Silencing Efficincy Evaluation of Free siRNA, siRNA@PEI@AuNP, and siRNA@PEI@SEMA@AuNP

**Figure 5:**
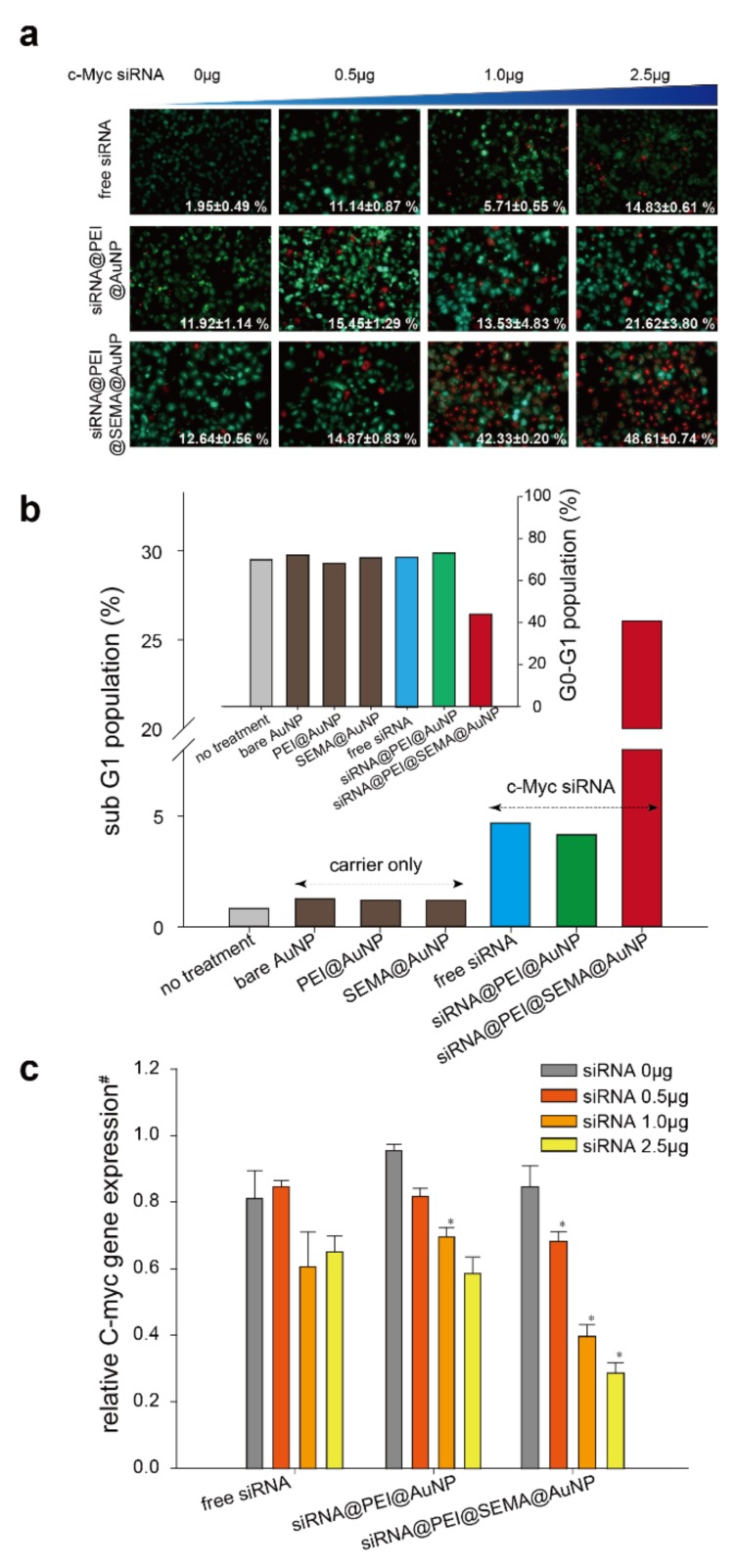
Anti-cancer effects of AuNPs. (**a**) LIVE/DEAD assay against A549 cells. Cells were stained by calcein AM (green) and ethidium homodimer-1 (red) for live and dead cells, respectively. Populations of dead cells out of total cells (100 cells) were image-analyzed to calculate populations of dead cells. At least three images were analyzed for each group and labeled in each image. (**b**) Cell population at sub-G1 phase was determined by flow cytometry to measure the apoptotic effect of siRNA@PEI@SEMA@AuNP. Inserted graph describes percentage of cells at G0-G1 phase. Histograms of flow cytometric analysis are shown in Figure S3. (**c**) C-myc gene expression level was measured by quantitative real-time PCR. #Normalized to GAPDH expression levels of free siRNA. * indicates a statistical significance in respect to free siRNA (*p* < 0.05).

To assess the anti-cancer effects of c-Myc siRNA incorporated AuNPs, we performed several cytotoxicity assays and apoptosis assays against A549 cells (Figure 5, Figure S2). The LIVE/DEAD cell assay results clearly indicated that PEI@SEMA@AuNP most strongly increased the gene transfection efficiency of c-Myc siRNA at the same amount of siRNA; dead cell populations of siRNA@PEI@SEMA@AuNP, siRNA@PEI@AuNP, and free siRNA with 2.5 μg of c-Myc siRNA were 48.61, 21.62, and 14.83%, respectively. The amount of dead cell population was not statistically different when the amount of siRNA was less than 1.0 μg. We therefore speculate that effects of PEI on the overall cytotoxicity of the carrier became more significant and overrode the cytotoxic effects of c-Myc siRNA. Further, when we compared the cytotoxicity of various AuNPs, PEI-incorporated AuNP exhibited considerable cytotoxicity, even in the absence of c-Myc siRNA (PEI@AuNP), while SEMA@AuNP was virtually non-toxic (Figure S2). Interestingly, the presence of the anionic corona on the AuNP contributed to decreasing the cytotoxicity of PEI (PEI@SEMA@AuNP). Because PEI has been considered to be cytotoxic at higher concentrations, the amount of PEI must be optimized to prevent undesirable cytotoxicity of gene carriers [25]. Considering that the release of PEI was significantly suppressed when pSEMA was decorated on the surface in comparison to bare AuNP (Figure S1), we speculate that the liberated PEI from the carrier contributed to the increase of cytotoxicity in PEI@AuNP. Thus, the surface decoration of pSEMA on AuNP reduced carrier-derived cytotoxicity of the gene carriers, while gene silencing effects of the incorporated c-Myc siRNA were not compromised [26,27]. To further elucidate the underlying mechanism of cell death, we analyzed the cell cycle arrest of the treated cells and determined the apoptotic death of the treated cells (Figure 5b) [28]. When a sub G1 cell cycle arrest was compared, siRNA@PEI@SEMA@AuNP exhibited the highest arrest rate among cells, which was followed by siRNA and siRNA@PEI@AuNP, consecutively; the cells treated with siRNA loaded PEI@SEMA@AuNP at extended early phases (sub G1) constituted 26.05% of the total phases analyzed, which was followed by cells with siRNA loaded PEI@AuNPs and free c-Myc siRNA (4.16% and 4.68%, respectively). Moreover, the analysis of cell population at G0–G1 significantly showed that siRNA@PEI@SEMA@AuNP induced apoptotic cell death, while cells with other AuNPs did not show noticeable changes of the cell cycle (Figure 5b-inset). Cell population at G0–G1 with siRNA@PEI@SEMA@AuNP exhibited decreased populations by 30~40% compared to other groups. Although cell populations at sub-G1 and G0–G1 are widely employed to determine apoptotic death, many reports indicate that the sub-G1 population can be more sensitive than the G0-G1 population in terms of evaluating apoptotic cell death [29]. These results indicate that pSEMA decorated AuNPs effectively deliver c-Myc siRNA to target cells because PEI can be highly incorporated onto the anionic corona of the AuNP and can consequently tightly bind and protect the siRNA. Previous reports indicated that siRNA can be easily degraded in culture medium, which considerably decreases the transfection efficiency of siRNA [30]. Thus, the anionic corona on AuNP can enhance PEI incorporation on the carrier, which allows siRNA to be efficiently incorporated and protected from degradation during the transfection process. Suppression of c-Myc was also evidenced using qRT-PCR, where siRNA@PEI@SEMA@AuNP exhibited the highest suppression of c-Myc (Figure 5c). While the carrier itself did not suppress the c-Myc expression, the suppression levels gradually increased when siRNA dose was increased from 0.5~2.5 μg in all samples. Although AuNP without the anionic corona (siRNA@PEI@AuNP) did not significantly suppress c-Myc expression in comparison to free siRNA, the presence of pSEMA clearly suppressed c-Myc expression with a statistical significance (siRNA@PEI@SEMA@AuNP). Specifically, when 2.5 μg of siRNA was employed, siRNA incorporated in PEI@SEMA@AuNP exhibited a 56% decrease of c-Myc expression in comparison to free siRNA transfection. These results indicate that pSEMA layers immobilized on the AuNP greatly enhance the transfection efficiency of siRNA, which consequently implies several rate determining steps during transfection can be facilitated by the anionic corona on the AuNP. We speculate that the liberation of siRNA from the carrier can be easily accomplished because pSEMA is linked to the surface of AuNP by Au-S linkages, which can be cleaved in reductive conditions, such as endosomes [31,32]. In fact, the similar digestions of Au-S linkages have been widely employed to control the release of incorporated drugs from Au-based nano-carriers [33,34,35]. They reported that polymer chains with reducible linkers were collapsed and cleaved in a cytoplasmic area, presumably in the endosome. Thus, the incorporated siRNAs in those carriers were liberated and finally combined with RISC to recognize and degrade the target c-Myc mRNA. Similarly, the pSEMA surrounding siRNA@PEI@SEMA@AuNP can be easily liberated from the AuNP by cleaving the Au-S linkers. Consequently, the liberated siRNA/PEI/pSEMA can have higher chances of escape from endosomes in comparison to PEI-immobilized AuNPs, whose PEI cannot be easily liberated.

### 3.4. In Vivo Anti-Cancer Efficincy Evaluation of Free siRNA and siRNA@PEI@SEMA@AuNP

**Figure 6:**
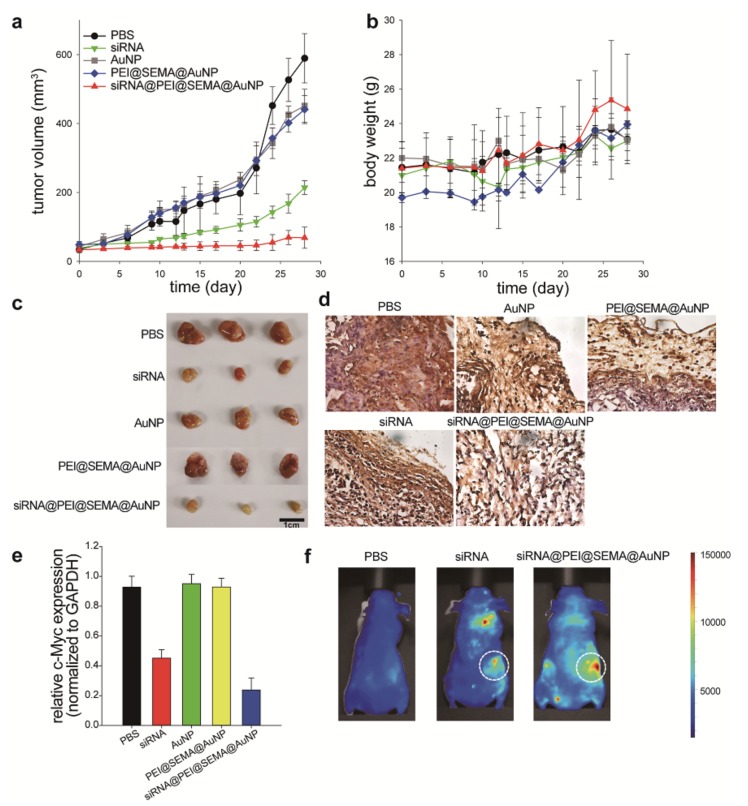
Animal study to determine in vivo anti-cancer effect of siRNA loaded AuNP to tumor-bearing mice. Intravenous injections were performed to the tail vein five times every 3 days. (**a**) Changes of tumor volume and (**b**) body weights were monitored for 28 days. The arrows at x-axis indicate the time of injection. (**c**) Tumors were harvested and compared for the size after 28 days. (**d**) Tumor tissues were visualized by immunohistochemical staining with anti c-Myc antibody (brown) and counter-staining with hematoxylin (purple). (**e**) Relative c-Myc gene suppression levels of the tumor tissues were determined by qRT-PCR. (**f**) Fluorescence imaging of live animals. Phosphate-buffered saline (PBS) and Alexa Fluor 647-labeled siRNA (AF647-siRNA) and AF647-siRNA@PEI@SEMA@AuNPs were injected, and the anesthetized animals were imaged after 12 h.

To confirm the in vivo efficacy and the bio-distribution of siRNA-loaded AuNPs, we intravenously injected these particles into tumor-bearing mice every 3 days for 12 days (Figure 6). During the monitoring of tumor volume changes for 28 days, the siRNA@PEI@SEMA@AuNP administered group exhibited the most dramatic suppression of tumor volume, while those with free siRNA, AuNP, carriers only, and PBS showed similar degrees of tumor size increase (Figure 6a). Specifically, at 28 days post administration, animals with siRNA@PEI@SEMA@AuNP (68 mm^3^) exhibited ca. 3.1-folds decrease of tumor volume in comparison to those with siRNA (214 mm^3^). Additionally, drug carriers without siRNA, such as PEI@SEMA@AuNP and AuNP, showed a similar volume of tumor size to that of an untreated group, suggesting that the carriers themselves would not produce noticeable anti-tumor effects. Furthermore, no noticeable change of the body weight was observed for all groups, which indicates that siRNA treatment did not cause any adverse effects (Figure 6b). We also harvested tumor tissues, re-confirmed the size of the tumors, and performed immune-histochemical staining against c-Myc and qRT-PCR for the isolated RNA from the tumor tissues to quantify the suppression levels of c-Myc at tumor sites (Figure 6c–e). Staining intensity against c-Myc protein was the most significant in the non-treated group (PBS), and the intensity was weak in the groups with AuNP, PEI@SEMA@AuNP, and siRNA (Figure 6d). However, tumors treated with siRNA@PEI@SEMA@AuNP exhibited the lowest intensity of anti-c-Myc staining, suggesting the most suppressed expression of c-Myc protein at the tumor sites. This was also evidenced by qRT-PCR of c-Myc mRNA, where the tumor tissues with siRNA@PEI@SEMA@AuNP showed 50% attenuated expression in comparison to those with free siRNA (Figure 6e). Animals with PBS or carriers (AuNP or PEI@SEMA@AuNP) exhibited similar levels of c-Myc expression at the tumor sites, clearly indicating that the carrier does not affect the suppression level of the oncogene. Previous studies accordingly showed that c-Myc suppression levels were closely associated with the suppression of tumor growth, which was based on both in vitro and in vivo works [36,37,38,39]. Thus, we anticipate that the c-Myc can serve as a marker indicating the successful suppression of tumor growth by silencing the c-myc gene with our siRNA-incorporated AuNPs; amongst the AuNPs, siRNA@PEI@SEMA@AuNP effectively silenced the c-myc gene and consequently inhibited tumor growth. Finally, we investigated the biodistribution of the siRNA-incorporated AuNP using fluorescence-tagging; after 12 h, considerable fluorescence intensity was visualized at the tumor sites (Figure 6f). Compared to animals with Alexa-siRNA, those with siRNA@PEI@SEMA@AuNP showed a highly-localized biodistribution of siRNA; however, siRNA was likely to be localized in both lung and tumors. Many studies accordingly indicate that intravenously-injected siRNA is attracted to lung tissues; thus, tumor localization is relatively limited, which explains why cationic gene carriers are employed to localize the therapeutic gene at tumor sites via an enhanced permeation and retention effect. Likewise, siRNA@PEI@SEMA@AuNP was shown to be attracted to tumor sites without noticeable localization at the lung tissue; this is advantageous to increase the anti-cancer efficacy of the gene carrier. Previous studies commonly employed AuNP for siRNA delivery, however, PEI decoration on AuNP was not efficiently accomplished because of the rigidity of the metal particles [40,41]. Because SEMA@AuNP exhibited higher retention of PEI on the surface in comparison to native AuNP, siRNA could be efficiently layered onto the PEI@SEMA@AuNP. Thus, we confirmed that a layer-by-layer assembly of siRNA and PEI on the anionic corona of AuNP can be a useful strategy to employ metallic nanoparticles for in vitro and in vivo gene delivery.

## 4. Conclusions

pSEMA was chemically synthesized on the surface of AuNPs to fabricate anionic corona for higher electrostatic interaction with PEI. siRNA@PEI@SEMA@AuNPs exhibited higher and more stable incorporation of siRNA in comparison to those without the pSEMA corona. In vitro and in vivo anti-cancer effects of siRNA@PEI@SEMA@AuNPs were demonstrated, and the anti-cancer effects caused by suppression of c-Myc expression at the tumor sites with higher localization of the AuNP were observed. Thus, we speculate that SI-ATRP based surface-decoration of AuNP can be tailored to incorporate cationic polymers and nucleic acids to metallic nanoparticles for gene delivery.

## Figures and Tables

**Table 1:** Size and surface charge of bare AuNPs and SEMA@AuNPs, PEI@SEMA@AuNPs, and PEI@AuNPs. All measurements were performed in triplicate manner, and each value was presented as average and standard deviation (*n* = 5).

Carriers	Diameter (nm)	ζ-Potential (mV)
bare AuNPs	25.55 ± 0.49	−23.87 ± 0.50
SEMA@AuNPs	222.20 ± 4.75	−26.55 ± 1.91
PEI@SEMA@AuNPs	161.08 ± 1.79	36.93 ± 0.97
PEI@AuNPs	180.63 ± 1.94	14.37 ± 1.94

## Supplementary Materials

The following are available online at https://www.mdpi.com/1999-4923/12/3/261/s1, Figure S1: Incorporated PEI on SEMA@AuNP or AuNP. AuNPs was incubated in PBS at 37 °C. The supernatant was collected and fluorescently labeled PEI was quantified by fluorescence spectroscopy, Figure S2: Cytotoxicities of AuNPs with or without siRNA by using MTT assay to A549 cell line. Cytotoxicities were measured by absorbance value with normalized to no treatment. PEI incorporated gold nanoparticles were conjugated with 2.5 µg siRNA, Figure S3. Cell cycles determined by flow cytometry with PI staining. All cycles (sub G1, G0–G1, S, G2–M) were expressed by stacking their percent.
