# Supplementary material for: Surface-Initiated Atom Transfer Polymerized Anionic Corona on Gold Nanoparticles for Anti-Cancer Therapy
Source: Pharmaceutics. 2020 Mar 13;12(3):261. doi: 10.3390/pharmaceutics12030261 (PMC7150926; doi:10.3390/pharmaceutics12030261)
Supplement: Supplementary file 1 [file pharmaceutics-12-00261-s001.pdf]

pharmaceutics

MDPI

Supplementary Materials: Surface-Initiated Atom Transfer Polymerized Anionic Corona on Gold Nanoparticles for Anti-Cancer Therapy

Wei Mao, Sol Lee, Ji Un Shin and Hyuk Sang Yoo

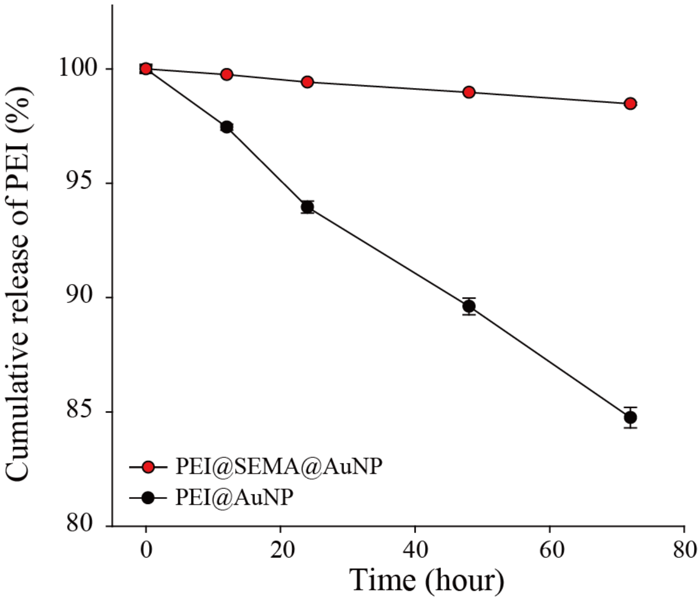

Figure S1. Incorporated PEI on SEMA@AuNP or AuNP. AuNPs was incubated in PBS at 37 °C. The supernatant was collected and fluorescently labeled PEI was quantified by fluorescence spectroscopy.

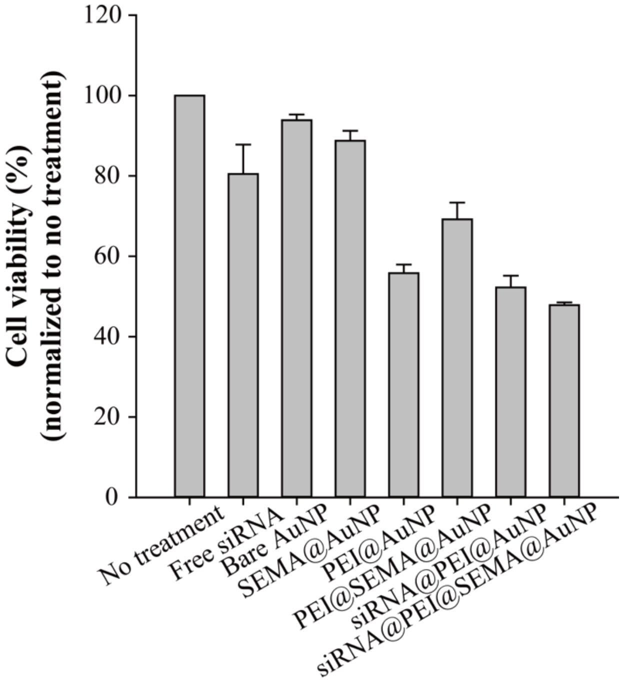

Figure S2. Cytotoxicities of AuNPs with or without siRNA by using MTT assay to A549 cell line. Cytotoxicities were measured by absorbance value with normalized to no treatment. PEI incorporated gold nanoparticles were conjugated with 2.5 μg siRNA.

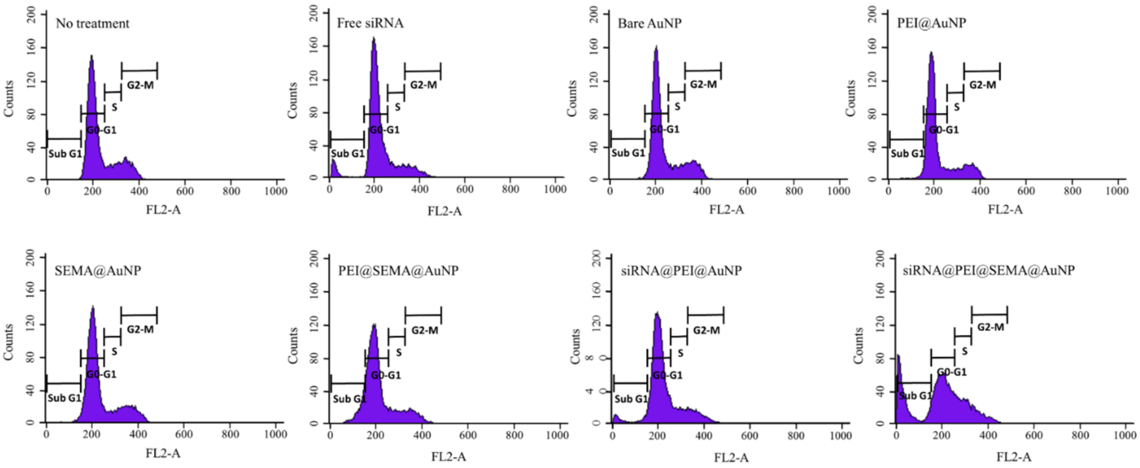

Figure S3. Cell cycles determined by flow cytometry with PI staining. Each cycle (sub G1, G0-G1, S, G2-M) were expressed by stacking their percent.
